# *ABCB1 *genotypes and haplotypes in patients with dementia and age-matched non-demented control patients

**DOI:** 10.1186/1750-1326-1-13

**Published:** 2006-09-25

**Authors:** Suzanne V Frankfort, Valerie D Doodeman, Remco Bakker, Linda R Tulner, Jos PCM van Campen, Paul HM Smits, Jos H Beijnen

**Affiliations:** 1 Department of Geriatric Medicine, Slotervaart Hospital, Amsterdam, The Netherlands; 2 Department of Pharmacy & Pharmacology, Slotervaart Hospital, Amsterdam, The Netherlands; 3 Department of Molecular Biology, Slotervaart Hospital, Amsterdam, The Netherlands; 4 Department of Medical Oncology, The Netherlands Cancer Institute/Antoni van Leeuwenhoek Hospital, Amsterdam, The Netherlands; 5 Faculty of Pharmaceutical Sciences, Department of Biomedical Analysis, Division of Drug Toxicology, Utrecht University, Utrecht, The Netherlands

## Abstract

Amyloid β is an *in vitro *substrate for P-glycoprotein (P-gp), an efflux pump at the blood brain barrier (BBB). The Multi Drug Resistance (*ABCB1*) gene, encoding for P-gp, is highly polymorphic and this may result in a changed function of P-gp and may possibly interfere with the pathogenesis of Alzheimer's disease. This study investigates to what extent *ABCB1 *Single Nucleotide Polymorphisms (SNPs; C1236T in exon 12, G2677T/A in exon 21 and C3435T in exon 26) and inferred haplotypes exist in an elderly population and if these SNPs and haplotypes differ between patients with dementia and age-matched non-demented control patients. *ABCB1 *genotype, allele and haplotype frequencies were neither significantly different between patients with dementia and age-matched controls, nor between subgroups of different types of dementia nor age-matched controls. This study shows *ABCB1 *genotype frequencies to be comparable with described younger populations. To our knowledge this is the first study on *ABCB1 *genotypes in dementia. *ABCB1 *genotypes are presently not useful as a biomarker for dementia, as they were not significantly different between demented patients and age-matched control subjects.

## Findings

P-glycoprotein (P-gp), a 170 kDa membrane bound efflux pump at the apical membrane of endothelial cells, functions as part of the blood brain barrier (BBB) [1,2] and is also expressed at the blood-cerebrospinal fluid (BCSF) barrier, formed by the choroid plexus [3]. The Multi Drug Resistance gene (*ABCB1*) encodes for P-gp. It is known that the *ABCB1 *gene is highly polymorphic [4]. The three most frequently occurring Single Nucleotide Polymorphisms (SNPs) are C1236T in exon 12 (dbSNP: rs1128503), G2677T/A in exon 21 (dbSNP: rs2032582) and C3435T in exon 26 (dbSNP: rs1045642) [5]. *ABCB1 *haplotypes composed of different SNPs may better represent changes in P-gp function [4].

The "amyloid hypothesis" states that accumulation of beta amyloid peptides in the brain is the key event in the pathogenesis of Alzheimer's Disease (AD) [6]. Amyloid deposits in plaques in brain parenchyma and along the vascular system [7]. Amyloid β is an *in vitro *substrate for P-gp [8] and recent research found that P-gp deficiency at the BBB increases β amyloid deposition in an AD mouse model [9]. Vogelgesang *et al*.[10] showed P-gp expression at the BBB to be inversely correlated to the number of amyloid plaques in the medial temporal lobe in 243 non-demented elderly. Thus, the efflux pump P-gp possibly plays a role in the pathogenesis of late-onset dementia by interfering with the amyloid clearance, as late-onset AD would result from inefficient clearance of beta amyloid from the brain [11].

We hypothesized *ABCB1 *genotypes to be related to dementia occurrence as amyloid load in the brain is possibly inversely related to P-gp expression at the BBB and *ABCB1 *SNPs and haplotypes may be related to P-gp expression and function. This study aimed to test this hypothesis in an elderly population consisting of patients suffering from dementia and age-matched non-demented control patients.

This prospective study was carried out at the geriatric diagnostic day-clinic of the Slotervaart Hospital, a teaching hospital in Amsterdam, the Netherlands. Dementia was diagnosed or excluded after performing complete geriatric assessment including: Mini Mental State Examination (MMSE) [12], the 7-Minute Neurocognitive Screening Test[13] and laboratory testing, including thyroid function, levels of folic acid, thiamine and vitamin B12. Thereafter, patients underwent more extensive neuropsychological assessment and, if necessary, computerized tomography or magnetic resonance imaging was performed. Mild Cognitive Impairment (MCI) and different types of dementia were diagnosed according to the current guidelines [14-19]. We categorized patients as unspecified dementia if the underlying process could not be diagnosed. Age-matched controls were recruited from the same diagnostic day-clinic. These participants did not show any cognitive impairment. Most of these geriatric patients were presented at the day clinic for a somatic screening. The study protocol was approved by the Institutional Review Board of the Slotervaart Hospital, Amsterdam, The Netherlands. Written informed consent was obtained from each participant in this study.

From each participant a 2 ml EDTA blood sample was obtained by venous puncture and genomic DNA was extracted using the Qiagen QIAamp^® ^DNA Mini Kit (Qiagen, Leusden, The Netherlands) according to the manufacturer protocol. *ABCB1 *was screened for C1236T in exon 12 (dbSNP: rs1128503), G2677T/A in exon 21 (dbSNP: rs2032582) and C3435T in exon 26 (dbSNP: rs1045642) by sequencing, as earlier described [20]. *APOE *genotype was determined using real-time polymerase chain reactions based upon Koch *et al*. [21].

The Chi square statistic test was used to calculate whether the alleles are in Hardy-Weinberg Equilibrium (HWE). Linkage disequilibrium (LD) between *ABCB1 *SNPs was performed by Graphical Overview of Linkage Disequilibrium (GOLD) software V1.1.0.0 [22] and haplotype analysis with the software package HPlus65v2.1.1 [23]. The Pearson Chi-square test was used to compare categorical variables and a one-way ANOVA for continuous variables. Kruskal-Wallis testing was performed to compare education level, scored on a seven-point scale, ranging from less than 6 years of elementary school (score 1) to a university degree (score 7) [24]. Logistic regression was performed to investigate the role of the *APOE *ε4 allele as a possible confounder, with the different types of dementia compared to controls as dependent variables and the described *ABCB1 *SNPs (as wild-type, heterozygous and homozygous mutants) and *APOE *(as ε4 allele carriers vs. non-carriers) as independent variables. A p-value of 0.05 or less was considered statistically significant. Bonferroni corrections were made in case of multiple testing. Statistical calculations were performed with SPSS for Windows (version 12.0, SPSS Inc., Chicago, IL, USA). A power analysis, using NQUERY advisor version 5.0, was performed for Chi-square testing between two groups (AD vs. controls) comparing proportions in three categories (wild-type, heterozygous and homozygous mutants).

In total, 161 patients signed informed consent. Seven patients were excluded, because of refusing further medical examination (n = 3) or because patients were diagnosed delirious (n = 4). The 154 included participants consisted of 113 patients (48 AD, 19 Vascular Dementia (VaD), 26 other dementia (OD), and 20 MCI) and 41 age-matched controls. The group of OD included 10 patients with a mixed type of dementia, 3 with Lewy Body Disease, 3 with alcohol induced dementia, 2 with Frontotemporal Dementia and 8 "unspecified" dementia syndromes. Baseline characteristics are presented in the table. The total population (n = 154) had a mean age of 81.7 ± 5.9 (63.3–94.8) years and almost 60% was female. Age, gender and education level were not significantly different between the subgroups. Mean MMSE score was significantly different between the dementia subgroups and the control group (p < 0.001), which is as expected.

Only in the MCI group allele frequencies for G2677T/A did not apply to Hardy Weinberg Equilibrium, although the other allele frequencies in all other groups did apply to HWE (results not shown). Strong linkage was observed between C1236T and G2677T/A (ρ^2 ^= 0.831, p < 0.000001), between C1236T and C3435T (ρ^2 ^= 0.424, p < 0.000001) and between G2677T/A and C3435T (ρ^2 ^= 0.456, p < 0.000001). In the table the *ABCB1 *genotype, allele and haplotype frequencies are presented. Haplotype data were inferred from genotype data only for Caucasian participants not possessing an A allele at position G2677T/A in exon 21. No statistical differences were observed for genotype data, allele frequencies and haplotype data between patients with dementia and age-matched controls, nor between patients with different types of dementia and age-matched controls. The logistic regression analyses did not show the APOE ε4 allele as confounder for the *ABCB1 *genotypes as possible risk factors for dementia (results not shown).

Frequencies of *ABCB1 *genotypes of the SNPs C1236T, G2677T/A, C3435T in this elderly population are comparable to earlier reports on younger populations [20,25,26]. We did not find a relation between *ABCB1 *SNPs and different types of dementia. Whether *ABCB1 *SNPs and haplotypes result in different function of P-gp at the BBB is not clear. In a study in 10 healthy volunteers who were homozygous for the TTT haplotype and in 10 healthy volunteers who where homozygous for the CGC haplotype, no differences in ^11^C-verapamil kinetics, as measured by Positron Emission Tomography, were apparent [27]. This could point out that *ABCB1 *SNPs and/or haplotypes are not related to P-gp function at the BBB.

This first study on *ABCB1 *genotypes in dementia has 27% power to detect differences in C3435T genotypes between AD and control patients. Based upon our preliminary results, 173 patients should be included in both the AD and the control group to obtain an ideally 80% power. This study and possible future ones may be combined in a meta-analysis to achieve more power to detect differences in *ABCB1 *genotypes between the different groups.

In conclusion, our study suggests that frequencies of *ABCB1 *genotypes and haplotypes are not significantly different between demented patients and age-matched control subjects and are presently not useful as biomarker for (different types of) dementia.

## Abbreviations

*ABCB1*: ATP-Binding Cassette Subfamily B member 1; *APOE*: Apolipoprotein E

## Competing interests

The author(s) declare that they have no competing interests.

## Authors' contributions

SF carried out design and interpretation of the study, included patients, participated in the sequence alignment, carried out statistical analyses, interpreted the results and drafted the manuscript. VD carried out the laboratory work. RB carried out the laboratory work. CT designed and interpreted the data, acquired data and helped to draft the manuscript. JvC designed and interpreted the data, included patients, helped to draft the manuscript. PS interpreted the data and reviewed the manuscript. JB participated in study design, statistics and interpretation and reviewed the manuscript. All authors read and approved the final manuscript.

**Table 1:** Demographic characteristics and *ABCB1 *genotype, allele and haplotype frequencies (n, (%))

	Total POP (n = 154)	Controls (n = 41)	AD (n = 48)	VaD (n = 19)	OD (n = 26)	MCI (n = 20)
Age, Mean ± SD (range)	81.7 ± 5.9 (63.3–94.8)	81.9 ± 5.7 (69.5–94.5)	81.0 ± 5.5 (71.9–93.3)	83.6 ± 5.6 (67.6–89.7)	82.1 ± 5.2 (69.6–94.8)	80.5 ± 6.9 (63.3–90.7)
Gender, n (%) female	92 (59.7)	27 (65.9)	33 (68.8)	8 (42.1)	11 (42.3)	13 (65.0)
Education, median IQR (range)	4 IQR: 3 (1–7)	4 IQR:3 (1–6)*	4 IQR:3 (1–7)^†^	5 IQR:2 (4–6)^†^	4 IQR: 3 (2–7)^†^	4 IQR:3 (2–6)*
Baseline MMSE, Mean ± SD (range)	21.2 ± 5.7 (3–30)	26.6 ± 2.2 (21–29)^¶^	18.2 ± 4.4^†,§ ^(6–26)^#^	18.1 ± 7.5^†,§ ^(3–29)	20.9 ± 5.2^† ^(12–30)^†^	25.3 ± 2.7 (19–30)^†^
Ethnicity, n (%) CAU	151 (98.1)	40 (97.6)	48 (100)	19 (100)	25 (96.2)	19 (95.0)

SNP C1236T (12)						
CC	44 (28.6)	12 (29.3)	12 (25.0)	3 (15.8)	7 (26.9)	10 (50.0)
CT	75 (48.7)	20 (48.8)	25 (52.1)	10 (52.6)	14 (53.8)	6 (30.0)
TT	35 (22.7)	9 (22.0)	11 (22.9)	6 (31.6)	5 (19.2)	4 (20.0)
Allele freq T (%)	0.471	0.463	0.490	0.579	0.462	0.350
SNP G2677T/A (21)						
GG	46 (29.9)	12 (29.3)	13 (27.1)	4 (21.2)	7 (26.9)	10 (50.0)
GT	67 (43.5)	16 (39.0)	24 (50.0)	9 (47.4)	13 (50.0)	5 (25.0)
TA	3 (1.9)	2 (4.9)	0 (0.0)	0 (0.0)	1 (3.8)	0 (0.0)
TT	38 (24.7)	11 (26.8)	11 (22.9)	6 (31.6)	5 (19.2)	5 (25.0)
Allele freq T (%)	0.474	0.488	0.479	0.553	0.462	0.375
Allele freq A (%)	0.010	0.024	0.000	0.000	0.019	0.000
SNP C3435T (26)						
CC	32 (20.8)	9 (22.0)	5 (10.4)	2 (10.5)	8 (30.8)	8 (40.0)
CT	70 (45.5)	18 (43.9)	26 (54.2)	9 (47.4)	10 (38.5)	7 (35.0)
TT	52 (33.8)	14 (34.1)	17 (35.4)	8 (42.1)	8 (30.8)	5 (25.0)
Allele freq T (%)	0.565	0.561	0.625	0.658	0.500	0.425

Haplotype	Total POP^a ^(n = 148)	Controls (n = 38)	AD (n = 48)	VaD (n = 19)	OD (n = 24)	MCI (n = 19)
T-T-T	130 (0.439)	33 (0.434)	43 (0.448)	20 (0.526)	22 (0.458)	12 (0.316)
C-G-C	114 (0.385)	29 (0.382)	34 (0.354)	11(0.289)	21 (0.438)	19 (0.500)
C-G-T	38 (0.128)	10 (0.132)	15 (0.156)	5 (0.132)	4 (0.083)	4 (0.105)
C-T-T	5 (0.017)	3 (0.039)	1 (0.010)	0 (0.000)	0 (0.000)	1 (0.026)
T-G-C	4 (0.014)	1 (0.013)	2 (0.021)	1 (0.026)	0 (0.000)	0 (0.000)
T-T-C	5 (0.017)	0 (0.000)	1 (0.001)	1 (0.026)	1 (0.021)	2 (0.053)

* missing for 2 patients. ^† ^missing for 1 patient. ^‡^p < 0.001 vs. controls. ^§^p < 0.001 vs. MCI ^¶^performed in 22 controls. ^#^missing for 3 patients.

Haplotype of the different SNPs, i.e. number of alleles and frequencies of the haplotype mentioned. C1236T-G2677T-C3435T;^a ^3 patients with an ethnicity different from Caucasian and 3 patients bearing an A allele at the 2677 (exon 21) position were excluded from haplotype analysis. POP = Population, AD = Alzheimer's Disease, VaD = Vascular Dementia, OD = Other dementias, MCI = Mild Cognitive Impairment, SD = Standard Deviation, IQR = Inter Quartile Range, MMSE = Mini Mental State Examination, CAU = Caucasian race, SNP = Single Nucleotide Polymorphism.
